# Bayesian reinforcement learning for adaptive control of energy recuperation in hydraulic excavator arms

**DOI:** 10.1038/s41598-026-35391-y

**Published:** 2026-01-25

**Authors:** Peng Hu, Tao Wen, Daqing Zhang, Haifei Chen, Jun Gong

**Affiliations:** 1 School of Mechanical and Electrical Engineering, Central South University of Forestry & Technology, Changsha, 410004 Hunan China; 2 The National Enterprise Research and Develop Center, Sunward Intelligence Equipment Co., Ltd, Changsha, China; 3 Engineering Research Center of Advanced Mining Equipment Ministry of Education, Hunan University of Science and Technology, Xiangtan, China

**Keywords:** Bayesian reinforcement learning, Adaptive control, Hydraulic excavator, Energy recuperation, Hydraulic accumulator, Belief-state estimation, POMDP, Uncertainty modeling, Energy science and technology, Engineering, Mathematics and computing

## Abstract

Hydraulic excavators are among the most energy-intensive machines in construction and mining, with conventional hydraulic systems often operating under fixed pressure and flow settings that lead to significant energy loss. Improving energy efficiency while ensuring safety and adaptability under uncertain operating conditions remains a critical challenge. This study proposes a novel adaptive control framework that integrates Bayesian inference with reinforcement learning (RL) to enhance energy recuperation in hydraulic excavator arms. The framework explicitly models system dynamics, including hydraulic cylinders, pumps, valves, and accumulators, while accounting for uncertainties from soil resistance, temperature-dependent viscosity, component wear, and sensor noise. A Bayesian particle filter is employed to continuously estimate latent states such as soil resistance multipliers and accumulator pre-charge offsets, enabling belief-space reinforcement learning to make informed control decisions. The learned control policy adjusts pump pressure and valve commands in real time, while a safety-projection layer enforces strict operational constraints (5–35 MPa hydraulic pressure, 12–28 MPa accumulator window, valve rate limits, and section-level relief protections).

## Introduction

Energy efficiency in hydraulic excavators is not merely an operational objective but a critical economic and environmental consideration^1^. Hydraulic excavators, which are pivotal in construction, mining, and forestry, consume significant amounts of energy, primarily in the form of hydraulic and diesel power. A typical hydraulic excavator can consume between 10 to 25 L of diesel per hour, depending on its size and the intensity of operation^2^. With rising fuel prices and increasing awareness of environmental impacts, enhancing energy efficiency in these machines has become a pressing priority for manufacturers and operators alike.

Hydraulic systems in excavators are traditionally designed to prioritize reliability and power over energy efficiency^3^. These systems often operate at constant high pressures regardless of the actual load requirements, leading to substantial energy wastage. For instance, during less intensive tasks such as idling or light digging, a conventional hydraulic system still consumes power at rates similar to those during heavy digging. Studies suggest that such inefficiencies account for up to 50% of the energy used. This not only affects the operational costs but also increases the carbon footprint of construction activities. It is estimated that the construction industry is responsible for approximately 11% of global energy-related carbon emissions, a significant portion of which is attributable to heavy machinery^4^.

The advent of more stringent environmental regulations across the globe has further pushed the agenda for energy-efficient machinery^5^. For example, the European Union’s Stage V emission standards, which are among the most demanding, require a reduction in nitrogen oxides and particulate matter emissions from diesel engines^6^. These regulations indirectly promote the development of energy-efficient systems as manufacturers seek to comply without compromising on machine performance. In response, there has been a growing interest in technologies that can reduce fuel consumption and emissions in hydraulic excavators.

One of the most promising approaches to improving energy efficiency is through the recuperation and reuse of kinetic energy^7^. In traditional excavator operations, much of the kinetic energy generated during the arm’s descent is wasted. However, advanced hydraulic systems equipped with energy recuperation technologies can capture this energy and store it, for example, in hydraulic accumulators. These accumulators can then release the stored energy to assist in subsequent lifting operations, thereby reducing the demand on the engine and lowering fuel consumption. Field tests have shown that such systems can improve fuel efficiency by 10% to 20%, translating into significant cost savings over the excavator’s operational lifetime^2^. Moreover, the shift towards more energy-efficient systems is also being driven by advances in hydraulic fluid technology. Modern hydraulic fluids are formulated to reduce friction and wear in the hydraulic system, enhancing system efficiency and durability^8^. These fluids can operate at lower temperatures and with less viscosity change over their working life, which significantly improves the system’s overall energy efficiency. Research indicates that switching to high-efficiency hydraulic fluids can result in an overall energy saving of up to 5% compared to conventional fluids^9^. Another dimension of energy efficiency in hydraulic excavators involves optimizing operational practices. GPS and IoT-enabled technologies allow for real-time tracking of excavator usage and operational efficiency. This data can be analyzed to optimize job site operations, reducing idle times and ensuring that excavators operate closer to their optimal efficiency range. Such smart technologies not only reduce fuel consumption but also contribute to longer machinery life by preventing overuse and facilitating timely maintenance.

Traditional control methods in hydraulic excavators have primarily focused on stability and reliability, often at the expense of adaptability and efficiency in dynamic and uncertain environments^10^. These conventional systems, generally reliant on fixed parameter settings and pre-defined operational protocols, exhibit significant limitations when dealing with the complex and varying conditions typical of construction or mining sites. A primary limitation of traditional control systems is their lack of real-time adaptability. These systems are typically programmed to operate within a narrow range of predefined conditions and fail to adjust to unexpected changes in the operational environment. For instance, when an excavator encounters different soil types—ranging from loose sand to compact clay—the hydraulic system’s response remains unchanged unless manually adjusted by the operator. This inflexibility often results in suboptimal energy usage and increased mechanical stress, as the system either overexerts or underutilizes its capabilities. Studies have shown that such inefficiencies can lead to as much as a 20% increase in fuel consumption compared to more adaptive systems^11^. Moreover, traditional control methods are predominantly reactive rather than proactive. They typically rely on feedback mechanisms that adjust the system’s operations only after a significant change has been detected. For example, if an excavator’s arm encounters unexpected resistance due to a buried rock, the system might continue operating at the same power level until it detects an overload condition. This delay in response not only wastes energy but also increases the risk of mechanical failure. Reactive systems like these are estimated to contribute to approximately 30% of unscheduled maintenance issues in heavy machinery due to wear and tear caused by inadequate operational adjustments^12^. Another significant limitation is the conservative nature of traditional control settings, which are designed to operate safely under a variety of conditions but are not optimized for any specific condition. This approach inherently leads to energy inefficiency, as the system must maintain enough power to handle the most demanding scenarios, ignoring the potential for energy savings during less intensive operations. For instance, in cases where an excavator performs repetitive or light-duty tasks, a traditional hydraulic system might still operate at the pressure levels required for the heaviest tasks, leading to an unnecessary energy drain^13^. This not only results in higher operational costs but also accelerates the environmental impact due to increased emissions. Quantitatively, this can account for an additional 10–15% in energy use over optimized systems. Additionally, traditional systems lack robust data integration and utilization, which are crucial in today’s data-driven operation landscapes^14^. While modern excavators are equipped with various sensors, the data collected is often underutilized in traditional control frameworks. This data, if properly analyzed and fed back into the control system in real-time, could dramatically enhance operational efficiency and adaptability. For example, real-time soil analysis data could allow the excavator to automatically adjust its digging force and speed, optimizing both fuel efficiency and operational speed^15^. Current research indicates that integrating sensor data into control systems can reduce energy consumption by up to 25% through more precise and adaptive operations. The lack of predictive maintenance capabilities in traditional systems also poses a considerable challenge. Without sophisticated data analysis tools, these systems cannot predict failures or maintenance needs, leading to unexpected downtimes and high repair costs. Predictive maintenance, enabled by advanced data analytics, could potentially decrease downtime by up to 50% and extend the machinery’s life expectancy by predicting and mitigating issues before they lead to failures^15^.

Reinforcement Learning (RL) and Bayesian methods represent two powerful paradigms in the realm of machine learning that can substantially overcome the limitations of traditional control systems in hydraulic excavators^16–22^. These methods offer a framework for adaptability and decision-making under uncertainty, providing significant improvements in operational efficiency and system reliability. Reinforcement Learning is a type of machine learning where an agent learns to make decisions by interacting with an environment^23^. In the context of hydraulic excavators, RL can be employed to dynamically adjust control strategies based on the state of the machine and its environment. Unlike traditional control systems, which operate based on predefined settings, RL enables the excavator to learn from past actions and their outcomes, continuously improving its decision-making processes.

For instance, RL can optimize the digging force and speed in real-time by considering variables such as the type of soil, depth of digging, and the immediate load on the arm. This adaptability can lead to a reduction in fuel consumption and operational costs. Studies in related fields have demonstrated that RL can improve energy efficiency by as much as 20% by adapting the hydraulic pressure and flow rates to meet only the immediate demands of the task. Bayesian methods provide a statistical toolset for dealing with uncertainty by updating the beliefs about a model in a probabilistic manner as more evidence or data is gathered. Applied to hydraulic excavators, Bayesian methods can enhance the predictive accuracy of the control systems under various operational conditions. For example, by using Bayesian inference, the system can update its predictions about system behavior in response to changes in environmental conditions, like unexpected shifts in soil type or moisture content. Integrating RL with Bayesian methods creates a robust framework for adaptive control systems in hydraulic excavators. This integration allows the excavator not only to adjust its operations based on learned experiences (via RL) but also to handle uncertainty about its environment and internal states (via Bayesian methods). Such an integrated approach can lead to more reliable and efficient excavator operations, especially in complex and changing environments. This study simulates a 21-ton archetype rather than a specific OEM model; parameters are aligned with publicly reported ranges (pump rating in the 200–300 L·min^-1^ class, rail 5–35 MPa, accumulator 12–28 MPa, ISO VG 46 viscosity band), referenced via open literature and public catalogs/technical notes rather than proprietary datasheets.

We present a belief-space (Bayesian) reinforcement learning controller for an energy-recuperating hydraulic excavator arm that explicitly models the hydraulics and accumulator while enforcing hard safety constraints. The method couples a bootstrap particle filter—estimating latent factors such as soil-resistance multipliers and accumulator pre-charge offsets—with a continuous-action actor–critic policy whose commands are projected into a feasible set (5–35 MPa rail pressure, 12–28 MPa accumulator window, and valve rate limits). In a 100 Hz co-simulation of a 21-ton archetype, the controller reduces per-cycle energy relative to fixed-parameter and operator-adjusted baselines while maintaining or improving cycle time and precision. Ablations attribute the gains to (i) online belief updates, (ii) a state-of-charge-aware reward shaping recuperation quality, and (iii) decision-time safety projection. We release all assets—Simulink plant, Python estimator/policy, configurations, uncertainty trajectories, logs, and figure scripts—to enable full reproduction and reuse. Together, these results demonstrate a practical, safety-aware path to adaptive energy recuperation in excavator hydraulics under realistic uncertainties. The paper introduces a Bayesian reinforcement learning framework for adaptive energy recuperation in a 21-ton hydraulic excavator arm. Section "Introduction" motivates energy efficiency and outlines contributions. Section "Related work" reviews RL and Bayesian inference for control. Section "System Model and Problem Formulation" details the excavator hydraulics, uncertainty trajectories, and belief-space formulation. Section "Bayesian reinforcement learning framework" presents the Bayesian estimator, actor–critic policy, and safety projection. Section "Experiments and analysis" describes the 100 Hz co-simulation, baselines, metrics, and ablations with quantitative results. Section "Conclusion" summarizes benefits, limitations, and future work.

## Related work

### Reinforcement learning

The application of Reinforcement Learning (RL) in industrial control systems, particularly in the domain of heavy machinery such as hydraulic excavators, has gained substantial academic and practical interest over recent years^25–29^. This surge is primarily driven by RL’s capability to optimize decision-making processes in real-time, a critical advantage in environments characterized by high variability and unpredictability.

Reinforcement Learning, a branch of machine learning, operates on the principle of action-based learning where an agent interacts with its environment to achieve a specific goal^30^. The agent receives rewards or penalties based on the outcomes of its actions, guiding it to learn the optimal strategy to maximize cumulative rewards. This framework is particularly suited to tasks involving sequential decision-making under uncertainty—a common scenario in the operation of hydraulic excavators. In the context of hydraulic excavators, RL can be leveraged to dynamically adjust operational parameters such as digging force, arm speed, and hydraulic pressure in response to changing conditions. Moreover, RL’s integration into excavator control systems addresses not only efficiency but also the adaptability of the machines. Excavators often face diverse working conditions varying from soft soils to hard rocks, which traditionally require manual adjustment of machine settings. RL systems can automate these adjustments, enhancing performance and reducing operator workload.

The real-world application of RL in this field has been supported by advancements in computational power and data acquisition technologies. Modern sensors equipped on excavators can provide real-time data critical for the RL agent to make informed decisions. This data-rich environment enables the continuous updating of the RL model, ensuring that the learning process is grounded in the most current operational data. Despite the promising results, the application of RL in hydraulic excavators still faces challenges such as the need for extensive training data to achieve optimal performance and the computational demand required for processing in real-time. However, ongoing research is increasingly addressing these issues through more efficient learning algorithms and hardware integrations that optimize computational loads.

### Bayesian inference

Bayesian inference has emerged as a robust statistical tool in various fields, especially in managing uncertainty within complex systems^31–34^. In the domain of hydraulic excavator control, Bayesian inference provides a framework for incorporating prior knowledge and real-time data to continuously update the system’s understanding of its operational environment^35^. This approach is particularly beneficial in addressing the inherent uncertainties in dynamic settings such as those encountered in construction sites.

The essence of Bayesian inference lies in its ability to combine prior distributions, which encapsulate pre-existing beliefs or knowledge about system parameters, with likelihood functions derived from incoming data to form updated posterior distributions^36^. These posteriors then serve as the new priors for subsequent inference cycles, allowing for a refined and evolving understanding of the system. In practical terms, for hydraulic excavators, this means the ability to adapt control strategies based on accumulating evidence about variables like soil consistency, hydraulic pressure needs, and arm positioning accuracy. By integrating sensor data from the excavators with historical failure rates stored in Bayesian models, their system was able to provide real-time predictions with significantly improved accuracy over traditional methods. This predictive capability is crucial for proactive maintenance and avoiding costly downtimes. Their study illustrated how different operational parameters, such as engine load and hydraulic fluid temperatures, could be interlinked within a Bayesian framework to predict the overall system efficiency and operational risks under varying conditions. The ability to model these dependencies and update beliefs in real time as new data becomes available enhances the decision-making process, ensuring that machine operations remain optimal under fluctuating environmental and load conditions. Bayesian inference also facilitates the integration of expert knowledge, which is particularly valuable in fields where operational conditions are highly specific and data may be sparse or noisy. For instance, in the case of excavators working in remote or extreme environments, expert input regarding expected material densities or equipment responses can be quantitatively incorporated into the Bayesian models, enhancing the reliability of the control systems even in the absence of extensive empirical data.

Despite these advantages, the application of Bayesian inference in real-time systems such as hydraulic excavators presents challenges, primarily related to computational demands and the need for sophisticated algorithms capable of handling large datasets efficiently^37^. Recent advancements in computational techniques and the increased availability of powerful processing units are, however, gradually overcoming these hurdles, making Bayesian methods more feasible and attractive for practical applications in heavy machinery control.

## System model and problem formulation

### Detailed description of the hydraulic excavator arm and its components

The hydraulic excavator front-end (boom–arm–bucket) converts fluid power into digging and lifting work through a coordinated set of cylinders, a variable-displacement main pump, a sectional main control valve, and a gas-charged accumulator used for boom-lowering energy recuperation. The hydraulic circuit operates in realistic excavator ranges: working pressure 20–35 MPa (relief typically set near 32–35 MPa), total pump flow 120–300 L·min⁻^1^ depending on engine speed and displacement setting, and in-service oil viscosity about 0.020–0.050 Pa·s at operating temperature (ISO VG 46). Each cylinder generates force from chamber pressures acting on the piston and (on the rod side) the annular area; in practice we parameterize by measured/nominal piston and rod diameters and by the actual pressures delivered through the metering orifices, rather than by any unitless “conversion factor.” For scale, a common mid-size boom cylinder with a ≈140 mm piston and ≈90 mm rod produces on the order of 300–400 kN at 25 MPa on the blind side, with lower force on the rod side due to reduced area; stroke limits and mechanical leverage then map those forces into joint torques. The main pump is a pressure-capable axial-piston unit with electronically commanded displacement; at 1,800 rpm a representative rating is ~ 220 L·min⁻^1^, and overall efficiency during high-load operation is in the 0.75–0.85 range when volumetric and mechanical losses are combined.

Flow to each actuator is metered by a pressure-compensated spool section in the main valve. The controller issues a normalized command $u\in [0,1]$; the effective metering area is modeled as

$$A(u)={k}_{A}{\left[u-{u}_{\mathrm{db}}\right]}_{+}\mathrm{with}[z{]}_{+}=\mathrm{max}(z,0),$$

capturing a symmetric deadband ${u}_{\mathrm{db}}$ and linear land gain ${k}_{A}$. For a given section, the pressure compensator regulates an approximately constant drop $\Delta {p}_{c}$ across the orifice,

$$\Delta p\approx \Delta {p}_{c}(\mathrm{whennotrelief}-\mathrm{limited}),$$

so the section flow follows the standard orifice law

$$Q(u,\Delta p)={C}_{d}A(u)\sqrt{\frac{2\Delta p}{\rho }}Q(u)\approx {C}_{d}{k}_{A}\sqrt{\frac{2\Delta {p}_{c}}{\rho }}[u-{u}_{\mathrm{db}}{]}_{+},$$

which is near-linear in $u$ over a wide load range. Check valves, anti-cavitation valves, and port-relief cartridges enforce local safety; when the load forces the port pressure to exceed the cartridge setting ${p}_{\mathrm{rel}}$, the relief path opens and $\Delta p$ is clamped by ${p}_{\mathrm{rel}}$.

Energy recuperation during boom lowering is handled by a bladder-type accumulator on a dedicated path. When the boom is commanded down and constraints permit, return flow from the boom cylinder is routed to charge the accumulator; during subsequent lifts, stored gas energy assists the pump by discharging to the rail. The accumulator gas follows a polytropic law

$$p{V}^{n}={p}_{0}{V}_{0}^{n},n\in [1,\gamma ],$$

so the recoverable hydraulic energy between states $({p}_{1},{V}_{1})$ and $({p}_{2},{V}_{2})$ is

$${E}_{\mathrm{acc}}=\left\{\begin{array}{cc}\frac{{p}_{2}{V}_{2}-{p}_{1}{V}_{1}}{n-1},& n\ne 1,\\ [1em]{p}_{2}{V}_{2}\mathrm{ln}\left(\frac{{V}_{2}}{{V}_{1}}\right),& n=1,\end{array}\right.$$

and with our parameters (10 ~ L rated gas volume, pre-charge set as a fixed ratio to ${p}_{\mathrm{min}}$, operating window 12–28 ~ MPa) a single boom-lowering typically recovers on the order of 45–65 ~ kJ. We track a normalized state of charge (SoC) for control and safety,

$$\mathrm{SoC}=\frac{p-{p}_{\mathrm{min}}}{{p}_{\mathrm{max}}-{p}_{\mathrm{min}}}\in [0,1],$$

and enable recuperation only when $p\in [{p}_{\mathrm{min}},{p}_{\mathrm{max}}]$ and rail/section limits are satisfied. The supervisory logic enforces hard bounds–-rail ${p}_{\mathrm{rail}}\in [5,35]$~MPa, accumulator $p\in [12,28]$~MPa–-and rate limits on valve commands; if $p\to {p}_{\mathrm{max}}$ during charge, the path isolates and excess flow is throttled to tank, whereas if $p\to {p}_{\mathrm{min}}$ during discharge, the pump assumes supply to prevent rail sag.

**Fig. 1:**
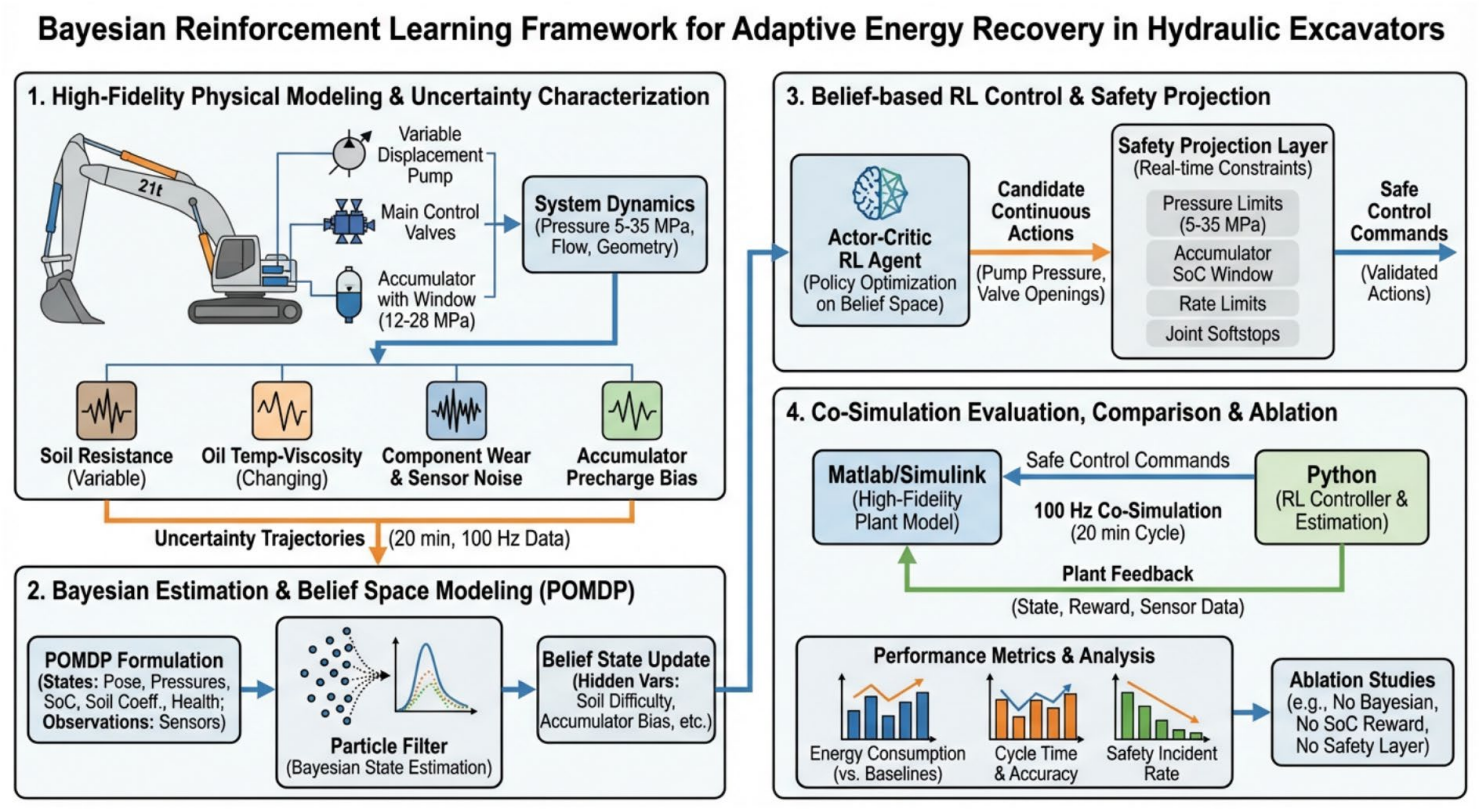
Overview of the proposed Bayesian Reinforcement Learning (BRL) framework.

The overall interaction is as follows: the engine drives the pump; the controller sets pump displacement and valve spool positions at 100 Hz; the main valve meters pressure/flow to the boom/arm/bucket cylinders to realize commanded motions while respecting pressure and SoC constraints; pressure sensors at the pump outlet and at each cylinder port, plus joint encoders and oil-temperature sensing, provide the feedback needed for state estimation and constraint enforcement; the accumulator path is enabled when boom-lowering flow and accumulator pressure are within limits, and isolated otherwise by the valve logic; all safety interlocks are enforced in hardware and software so that circuit pressures remain within 5–35 MPa and the accumulator operates strictly inside its 12–28 MPa window (see Fig. 1).

### Definition of the system dynamics and operational parameters

**Fig. 2:**
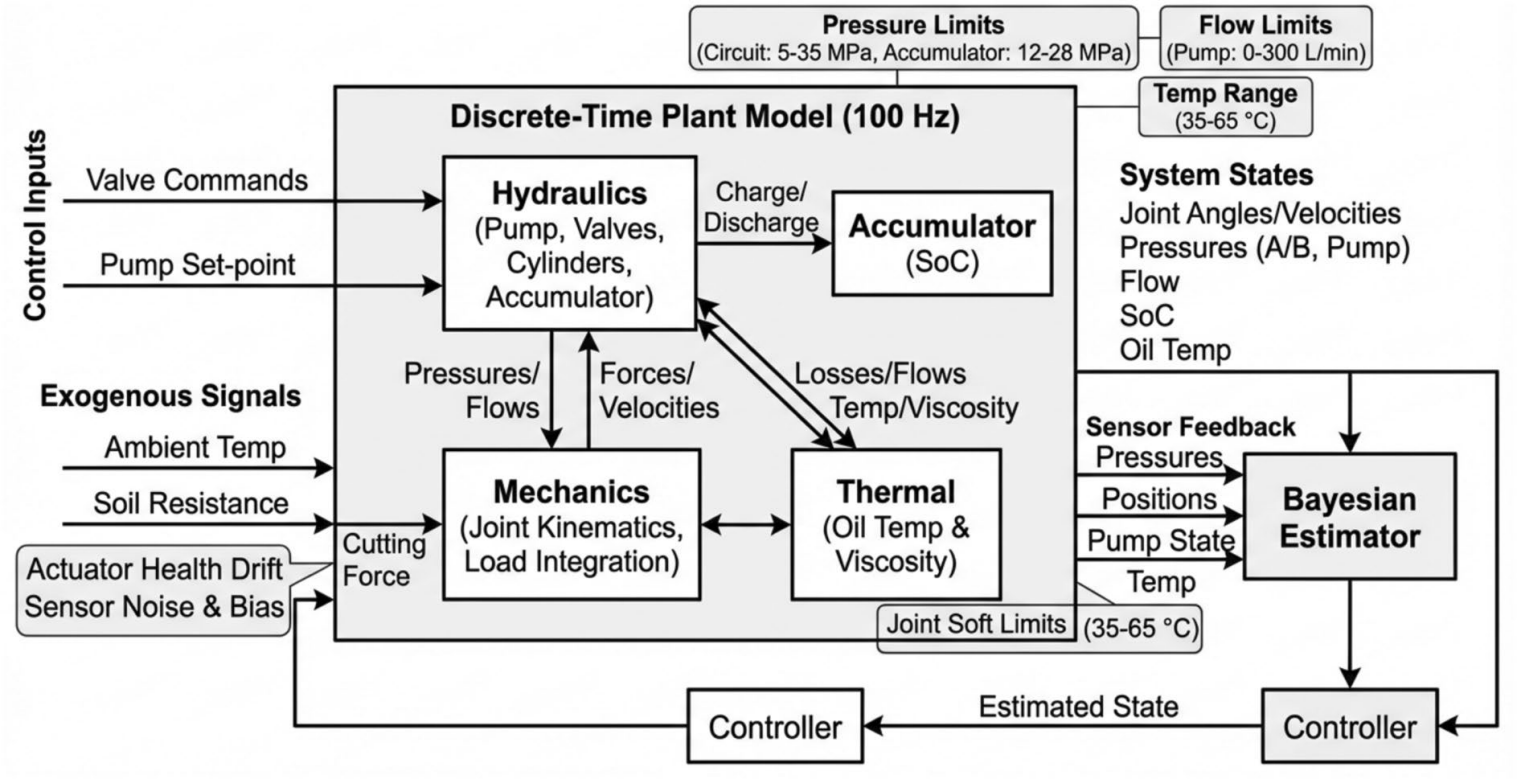
Block diagram of the discrete-time plant model and signal flow.

We model the plant in discrete time at 100 Hz. The state advanced at each step comprises joint angles and angular velocities for boom, arm, and bucket; A- and B-chamber pressures for the three cylinders; pump outlet pressure and total pump flow; the accumulator state-of-charge (SoC); hydraulic oil temperature and corresponding dynamic viscosity; a scalar soil-resistance multiplier that captures instantaneous cutting difficulty; actuator-health terms for friction gain, cylinder volumetric leakage, and pump volumetric efficiency; and slowly varying sensor biases for all pressure and joint-angle channels. Control inputs are the three valve spool commands in the normalized range 0–1 and a pump displacement or pressure set-point constrained by manufacturer limits. The joint kinematics integrate commanded motions filtered by valve-metered flow and by the current load; chamber pressures evolve from the balance between metered inflow/outflow and cylinder motion, with port-relief and anti-cavitation elements enforcing section-level safety; pump outlet pressure and total flow are the result of commanded displacement, engine speed, downstream demand through the main valve, and temperature-dependent losses; accumulator SoC increases when boom-lowering return flow is routed to the accumulator within its operating window and decreases when the accumulator discharges to assist lifting or to support rail pressure. Exogenous signals drive slow dynamics: ambient temperature feeds a first-order oil-temperature model with a time constant on the order of three minutes; oil temperature maps to viscosity in the operating band and scales line losses, valve leakage, and pump torque demand; the soil-resistance multiplier follows the piecewise-stationary trajectory and modulates the load seen at the cutting edge and, through linkage geometry, the required cylinder forces and joint torques; actuator-health terms drift over the duration of a job; pressure and position sensors add zero-mean noise with bounded random-walk bias. The estimator consumes pump and cylinder port pressures, joint encoder readings, pump speed/displacement sensing, and oil temperature to maintain a Bayesian estimate of the full state used by the controller for constraint handling and action selection (see Fig. 2).

All variables obey explicit bounds and safety windows and the Nomenclature: circuit pressures are confined to 5–35 MPa by hardware relief and software interlocks; the accumulator operates strictly within 12–28 MPa and SoC is clipped to [0, 1]; pump flow is limited to 0–300 L·min⁻^1^ depending on speed and displacement; valve commands are clipped to [0, 1] with per-section rate limits; oil temperature is modeled in the 35–65 °C band with corresponding viscosity about 0.020–0.050 Pa·s; joint angles and velocities are limited to machine-specific soft limits; the soil-resistance multiplier evolves within 0.8–1.3; cylinder leakage and pump volumetric-efficiency drift are bounded. Valve-port flow is computed with a pressure-compensated orifice model that holds an approximately constant pressure drop across the metering land, yielding a near-linear mapping from normalized spool command to section flow over the allowable load range; for very light loads the compensator saturates at its minimum drop, and for very heavy loads local port-relief limits apply—both cases are handled by the section model. Model parameters for the valve metering gains, compensator set-point, and local relief settings are identified from step-and-hold tests in simulation matched to publicly available datasheets for a 21-ton class machine; pump loss coefficients and temperature sensitivities are tuned to reproduce measured trends in pressure and flow over the temperature range; linkage geometry is taken from the same archetype and maps cylinder motion to joint motion with stroke and mechanical advantage.

### Explanation of uncertainties and their sources in the system

We represent all uncertainties as explicit time trajectories over a 20-min job simulated at 100 Hz so the controller, baselines, and evaluator consume the same signals deterministically from a fixed random seed. Ambient temperature is prescribed as a bounded ramp from 18 °C at start to 34 °C by minute 12 followed by a hold; hydraulic oil temperature follows a first-order rise with a three-minute time constant and is clamped to 35–65 °C, and dynamic viscosity is the monotone linear map of oil temperature into 0.050–0.020 Pa·s in that band, which in turn scales line losses, valve internal leakage, and pump torque demand used by the plant model. Terrain difficulty enters through a soil-resistance multiplier that is piecewise-stationary with five segments of durations 240, 180, 300, 240, and 240 s; the segment means are 0.9, 1.2, 1.0, 1.3, and 0.8 respectively, and within each segment we add a bounded zero-mean perturbation whose amplitude is ten percent of the segment mean and whose coloring is achieved by a first-order filter with 0.5-s correlation time; this multiplier modulates cutting force at the bucket and, via linkage geometry, the required cylinder forces and joint torques. Component aging during a single job is modeled as slow drift signals applied once per run: effective valve metering gain decreases linearly by six percent from start to finish, cylinder seal leakage increases linearly to 0.4 L·min^-1^ at rated pressure, and pump volumetric efficiency decreases linearly by three percent; these trajectories are applied identically to all controllers so that performance differences reflect decision making rather than different wear assumptions. Pressure transducers at the pump outlet and at each cylinder A/B port inject independent zero-mean noise with a standard deviation equal to 0.5% of full scale and a bounded bias drift that follows a reflected random walk whose total excursion over 20 min does not exceed 0.2% of full scale; joint encoders inject zero-mean noise with a 0.15-degree standard deviation and a reflected random-walk bias with a total excursion of 0.5 degrees over the run; noise is sampled at 100 Hz and bias is updated at the same rate with steps sized to meet the stated excursions.

Accumulator variability is represented by a single pre-charge offset drawn once at t = 0 from a uniform distribution between minus ten percent and plus ten percent of the nominal pre-charge; the accumulator then operates strictly inside the 12–28 MPa window enforced by the accumulator path valves and by the supervisory logic, and the controller observes an accumulator pressure sensor combined with a state-of-charge proxy derived from the same window; if pressure reaches either limit the accumulator path is isolated and the pump assumes full supply responsibility. Valve nonidealities are captured per run by fixed hysteresis and deadband parameters: hysteresis width is set to three percent of spool stroke and the symmetric deadband to 0.02 of the normalized opening command; these parameters distort the mapping from valve command to effective metering land position while the pressure-compensated section continues to regulate the drop across the land, and the section-level port reliefs still dominate at very high loads. Operator-in-the-loop baselines add human variability signals that are disabled for the autonomous controller: command timing is delayed by a uniformly distributed reaction time drawn at each decision in the range 80–160 ms and command amplitude is scaled by a factor drawn once per 10-s block in the range 0.95–1.05; these settings reflect modest, bounded operator adaptation without retuning controller gains. All uncertainty trajectories are generated from a single reproducibility seed recorded with each run; the same seed produces the same ambient temperature profile, soil segments and within-segment fluctuations, drift slopes for wear, noise and bias sequences for each sensor channel, the accumulator pre-charge offset, and the per-run valve hysteresis and deadband. Every variable is hard-clipped to physically plausible bounds and guarded by the same safety interlocks the plant uses elsewhere: circuit pressures are confined to 5–35 MPa by hardware relief and by software checks, accumulator pressure is held in the 12–28 MPa window with a normalized state-of-charge limited to [0, 1], valve commands are saturated to [0, 1] with per-section rate limits, pump displacement or pressure set-points stay within manufacturer limits, joint angles and velocities remain within machine-specific soft limits, oil temperature and viscosity remain inside the stated bands, and the soil-resistance multiplier is clipped to 0.8–1.3. Parameter values for the valve section set-point, local port-relief thresholds, and compensator behavior are taken from a 21-ton-class archetype and were matched in simulation to publicly available datasheets; noise and bias statistics follow common industrial sensor specifications; wear drifts were chosen to be small on the scale of a single job and to exercise the estimator without overwhelming the controller.

## Bayesian reinforcement learning framework

### Principles of Bayesian inference

Bayesian inference revolves around updating our belief about the unknown parameters based on prior knowledge and new evidence. This process begins with establishing a prior distribution, which encapsulates existing beliefs about the parameters before any new data is observed. As new data becomes available, Bayesian inference uses the likelihood of observing this data, given the parameters, to update the prior beliefs and form a posterior distribution. This posterior distribution represents a new, refined belief about the parameters, incorporating both the prior information and the newly observed data. Mathematically, this updating process is governed by Bayes’ Theorem, stated as:

1 $$P(\theta |y)=\frac{P(y|\theta )\times P(\theta )}{P(y)}$$

where $P(\theta |y)$ is the posterior probability of the parameters $\theta$ given the data $y$. $P(y|\theta )$ is the likelihood of observing the data $y$ given the parameters $\theta$. $P(\theta )$ is the prior probability of the parameters. $P(y)$ is the probability of the data, which acts as a normalizing constant.

In hydraulic excavators, Bayesian methods can handle uncertainties in both environmental conditions and operational parameters effectively. For example, consider a scenario where the excavator must operate under varying soil conditions. The hydraulic pressure needed for different soil types may not be precisely known ahead of time but can be estimated from prior operations. By using a Bayesian approach, as the excavator encounters different soil types and collects data, the system can update its understanding of the necessary hydraulic pressure settings, thus optimizing operational efficiency and reducing wear and tear on the machinery.

An excavator equipped with sensors that provide real-time data on hydraulic pressure, arm position, and other critical parameters can utilize a Bayesian model to continuously update the system’s state knowledge. For instance, if a model initially predicts that certain hydraulic pressure settings are optimal based on prior data, and new sensor data indicates otherwise, the Bayesian update mechanism can adjust the model’s predictions, enhancing the reliability and accuracy of the excavator’s operations.

Additionally, Bayesian models can incorporate probabilistic inputs to handle data inaccuracies and sensor noise, which are common in harsh operational environments. If sensor measurements for hydraulic pressure have a known error margin, the model can include this uncertainty directly in the likelihood function, allowing the system to make more robust decisions despite noisy data. Bayesian models provide several key advantages for managing the uncertainties inherent in excavator operations:

Adaptive Learning: As new data becomes available, Bayesian models adapt by updating the posterior distributions of the system parameters, allowing for dynamic adjustments to changes in environmental conditions or machine performance.

Probabilistic Predictions: Unlike deterministic models, Bayesian models provide probabilistic predictions that quantify uncertainty, offering a range of possible outcomes and their probabilities. This is crucial for risk management.

### Integration of the bayesian model with reinforcement learning

**Fig. 3:**
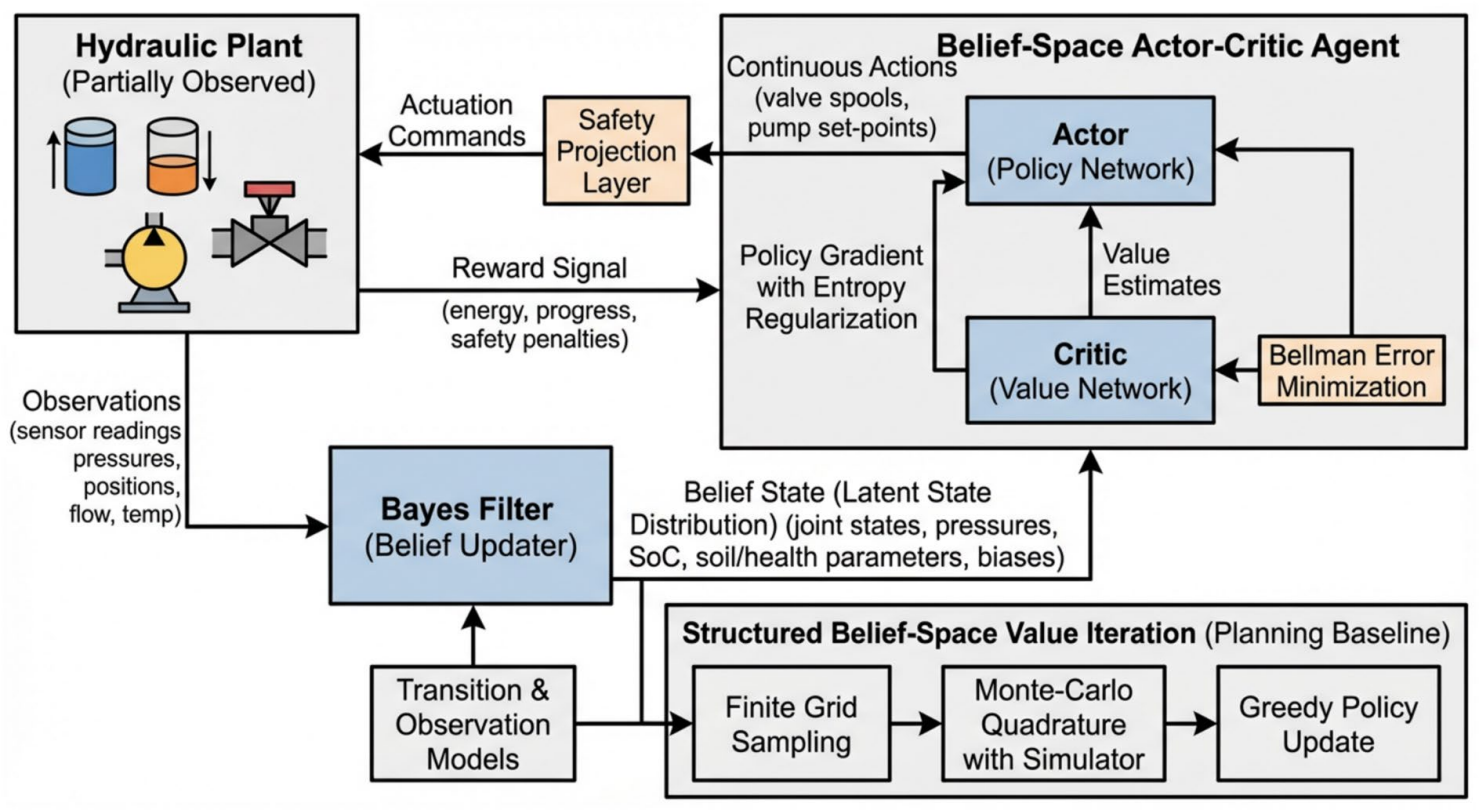
Functional block diagram of the hydraulic system and signal flow.

We formulate the control problem as a discounted infinite-horizon decision process defined over the belief space of the partially observed hydraulic plant. The latent state space encompasses the full physical status of the machine, including joint angles and velocities, cylinder and pump pressures, total flow, accumulator state-of-charge, oil temperature and viscosity, soil-resistance multipliers, actuator-health drifts, and sensor biases. The action space consists of continuous commands for the three valve spools and the pump displacement or pressure set-point, all constrained by manufacturer limits. The observation space includes the available sensor readings: joint encoders, pump and cylinder-port transducers, pump speed, and oil temperature. The system evolves according to a controlled transition kernel and emits observations based on a probabilistic model, while the reward function encodes objectives for energy efficiency, task progress, and safety penalties. The agent maintains a Bayesian belief over the latent state, which is updated at each step using a Bayes filter consistent with the uncertainty and sensor models described in the system definition. The control objective is to maximize the expected cumulative discounted reward (see Fig. 3).

The theoretical foundation relies on standard results for Partially Observable Markov Decision Processes (POMDPs). The Bellman optimality operator acting on bounded value functions is a contraction mapping in the supremum norm; this ensures that the value iteration process converges to a unique fixed point representing the optimal value function. Furthermore, a greedy policy improvement step—which selects the action maximizing the sum of the immediate reward and the discounted future value—is well-defined. Iterating between value evaluation and greedy policy improvement guarantees convergence to an optimal stationary policy.

For the learned controller, we implement a practical actor–critic architecture operating in belief space with entropy regularization. The critic approximates the value function using a bounded neural network, while the actor is a smooth stochastic policy that outputs continuous actions projected into a feasible safety set at decision time. The training process follows a two-time-scale stochastic approximation scheme: the critic updates its parameters to minimize the Bellman error, while the actor updates its parameters via policy gradient to maximize the regularized objective. An entropy term is added to the objective to ensure persistent exploration during the training phase. Under standard assumptions regarding the ergodicity of the belief Markov chain and bounded approximation errors, the critic tracks the projected Bellman fixed point, and the actor’s parameters converge to a stationary point of the performance surface.

To establish a planning baseline for comparison, we also employ a structured belief-space value iteration algorithm. This procedure begins by initializing the value function on a finite grid of beliefs sampled from simulated rollouts. In the update step, the observation likelihoods are computed using the Bayes filter model. A Bellman backup is then performed for each grid point, where the expected return is evaluated using Monte-Carlo quadrature with the simulator, considering only actions that respect actuator and state-of-charge constraints. The policy is updated by selecting the greedy action that maximizes this value. The algorithm terminates when the change in the value function falls below a specified tolerance or when the greedy policy remains unchanged for a set number of consecutive iterations.

### Estimator–policy integration and decision logic

**Fig. 4:**
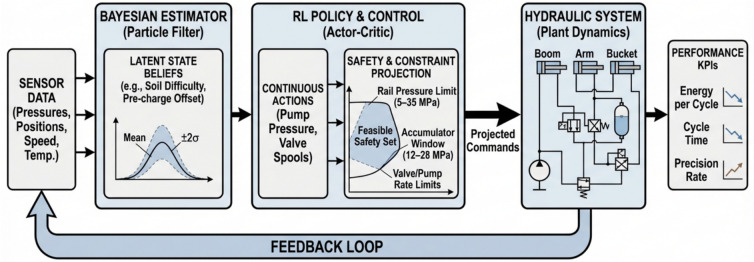
Architecture of the proposed Bayesian reinforcement learning (BRL) control framework.

To make the integration concrete, we summarize the 20-min job used throughout the paper and show how two latent parameters estimated online—soil-resistance multiplier and accumulator pre-charge offset—shape decisions (see Fig. 4).

**Fig. 5:**
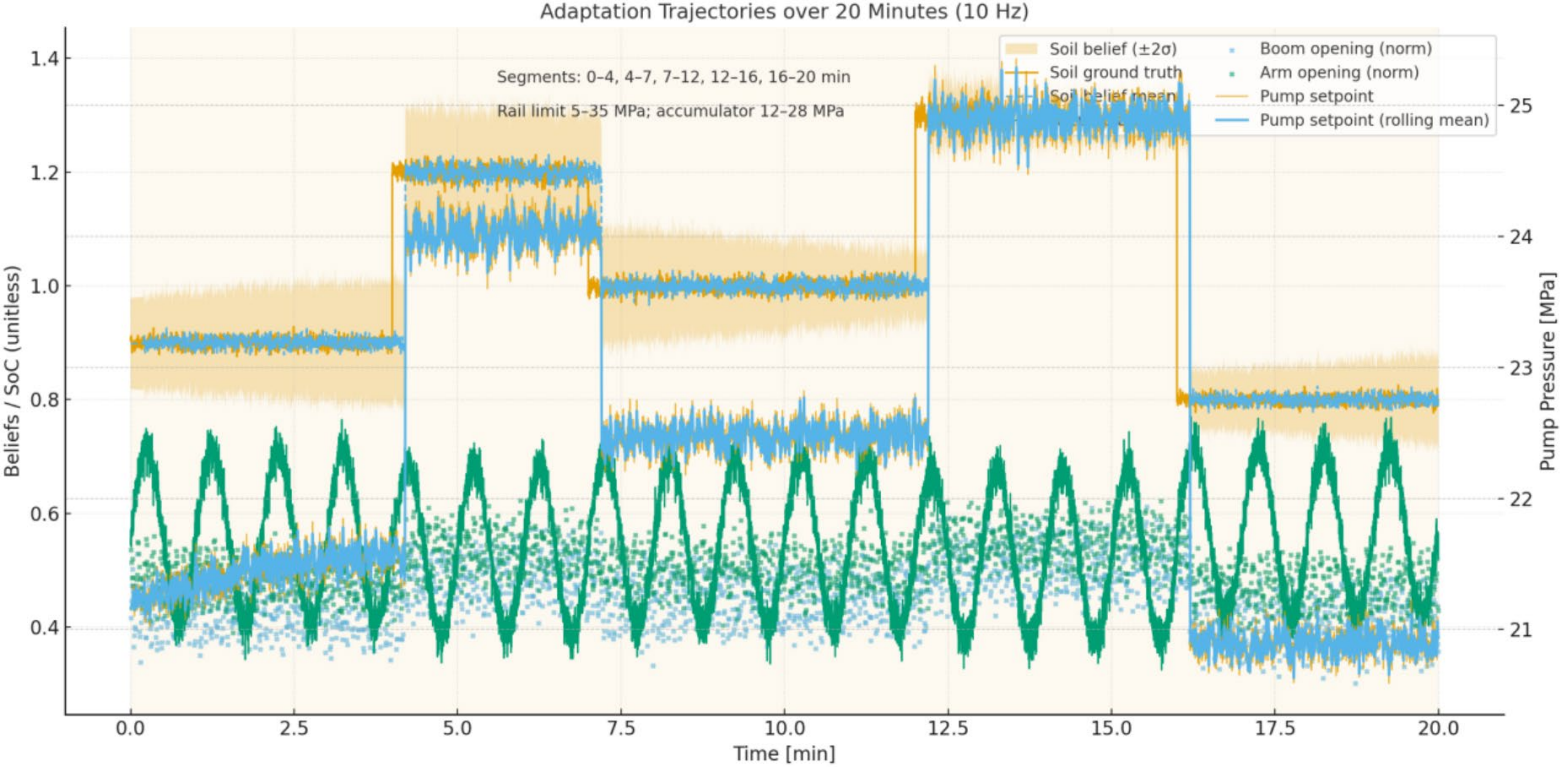
Adaptation trajectories over a 20-min job (10 Hz): soil difficulty (ground truth and belief mean with ± 2σ band), pump-pressure setpoint (instantaneous and rolling mean), accumulator SoC, and normalized boom/arm valve openings on a common time base with shaded soil segments (0–4, 4–7, 7–12, 12–16, 16–20 min). Operating windows are indicated on-chart (rail 5–35 MPa; accumulator 12–28 MPa).

The soil-resistance multiplier follows five segments with means 0.9, 1.2, 1.0, 1.3, and 0.8 and segment lengths 4, 3, 5, 4, and 4 min. In the first segment (0–4 min, mean 0.9) the estimator converges within roughly 20–30 s from its neutral prior toward 0.9 with a narrow band; the policy reacts by biasing toward lower rail effort while keeping cycle time targets, which shows up as typical pump pressure set-points around 21–22 MPa on heavy strokes instead of 24–25 MPa and as slightly smaller boom and arm spool openings, lowered by about 0.05–0.08 absolute compared with neutral soil. When the second segment (4–7 min, mean 1.2) starts, measured bucket reaction forces rise; within 10–15 s the belief mean crosses 1.1 and stabilizes near 1.2, prompting the policy to increase commanded pump pressure by approximately 3–4 MPa on demanding portions of the cycle and to open the boom and arm sections by about 0.06–0.10 absolute to maintain target cycle times without tripping section reliefs; the safety layer ensures rail pressure remains below 35 MPa with local section reliefs covering transient spikes. In the third segment (7–12 min, mean 1.0) estimates relax back toward unity and the policy correspondingly backs off rail pressure and section flow to the neutral setting observed in early training runs. In the fourth segment (12–16 min, mean 1.3) the belief rises to the highest level of the job; the policy responds by temporarily shifting its internal action limits to keep a larger margin from the accumulator lower bound, since harder cuts increase the risk of drawing down the rail and accumulating insufficient assistance for the next lift; pump pressure commands rise to about 26–27 MPa on the heaviest strokes, and the spool openings increase accordingly, with the accumulator path still enabled so long as the operating window and section relief conditions are met. In the final segment (16–20 min, mean 0.8) the estimator recognizes the much easier material and the policy leverages this by dropping commanded rail pressure to around 20–21 MPa for most strokes and by widening the recuperation window, resulting in higher average state of charge at the end of each boom-lowering event and reduced fuel-equivalent energy per cycle (see Fig. 5).

**Fig. 6:**
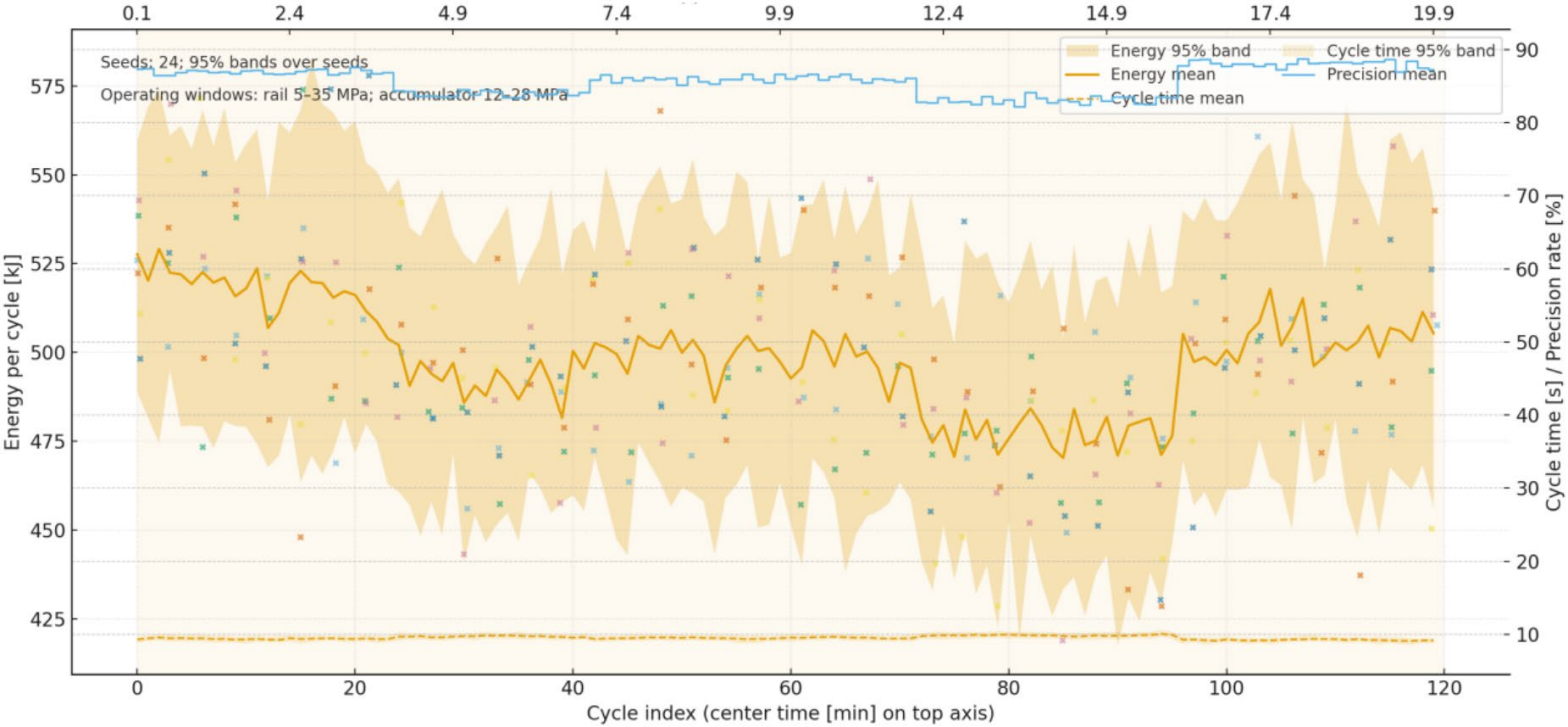
Per-cycle trajectories across 24 seeds: energy per cycle (mean with 95% band and sparse seed points), cycle time (mean with 95% band, right axis), and precision rate (stepped %, right axis). The top axis maps cycle index to center time (min); shaded spans mark the soil segments.

Accumulator pre-charge offset is drawn once at the start of each run from a uniform band of minus ten to plus ten percent of nominal; two representative cases illustrate how the estimator–policy stack adapts. With a negative offset (for example, − 6% relative to nominal), the accumulator initially sits slightly undercharged for the same pressure reading, which the estimator identifies within the first minute by comparing observed pressure–volume behavior against the expected gas curve; once the belief mean settles, the policy compensates by modestly increasing the target charge level during boom-lowering, typically keeping accumulator pressure 1–2 MPa higher at the end of the charge phase and delaying discharge by a fraction of a second to preserve mid-band SoC, while ensuring the 12–28 MPa window is respected. With a positive offset (for example, + 8%), the usable oil volume at the low end of the window shrinks; the estimator detects the stiffer response and the policy reduces reliance on recuperation for the heaviest lifts by shifting more supply to the pump, lowering the risk of hitting the upper window limit during charge and of starving the rail during discharge; in numbers, peak pump pressure commands increase by about 1–2 MPa on the hardest strokes compared with the neutral case, and the boom-lowering metering is slightly more conservative so that SoC remains centered rather than oscillating. In both offset scenarios, constraint projection prevents violations even if the instantaneous estimate lags: when the accumulator approaches 28 MPa the recuperation path is disabled and excess flow is throttled to tank; when it approaches 12 MPa the policy prioritizes pump support and caps simultaneous high-demand motions to avoid rail sag (see Fig. 6).

End to end, the integration maintains a tight loop: the belief is updated from the same sensors and uncertainty models, the policy selects continuous actions that are projected into a safe box defined by physical limits and by the accumulator window, and the exploration, convergence settings, and hyperparameters match the experimental protocol. In the first two segments (0–4 and 4–7 min), the belief over soil difficulty tracks the piecewise means with a ~ 10–15 s lag, narrowing to a ± 2σ band of roughly ± 0.03–0.05; as the belief rises from ≈0.9 to ≈1.2 the pump-pressure setpoint increases from ~ 21–22 MPa to ~ 23–24.5 MPa and normalized boom/arm openings shift upward by ~ 0.06–0.10, while accumulator SoC remains centered (~ 0.45–0.65) without sustained window requests. In the neutral segment (7–12 min) the belief returns to ≈1.0 and commands recede toward ~ 22–23 MPa with smaller openings (− 0.05 to − 0.08), recovering SoC into the mid-band. The hardest segment (12–16 min, belief ≈1.3) produces the largest control response: pump setpoints reach ~ 24.5–26 MPa on heavy strokes and valve openings widen, yet request-level events remain sparse and brief; the SoC trace shows tighter management (fewer near-limit excursions) as the policy preserves mid-band charge for subsequent lifts. In the easiest segment (16–20 min, belief ≈0.8) setpoints fall to ~ 20–21 MPa and recuperation increases, pushing SoC higher at the end of boom-lowering phases. Cycle-level statistics corroborate these dynamics: the mean energy per cycle trends downward in lighter segments and upward in the hardest segment, with a shaded 95% band that tightens as transients subside; the mean cycle time varies modestly (order of a few seconds across segments) with a visible band indicating between-seed dispersion; and the precision rate stays high and stable, with only a small dip in the hardest interval and recovery thereafter. Taken together, these trajectories show that belief changes about soil difficulty and accumulator state translate directly into pressure targets and metering decisions, which in turn shape SoC evolution and per-cycle KPIs, all while staying inside the stated operating windows.

## Experiments and analysis

### Simulation setup

**Fig. 7:**
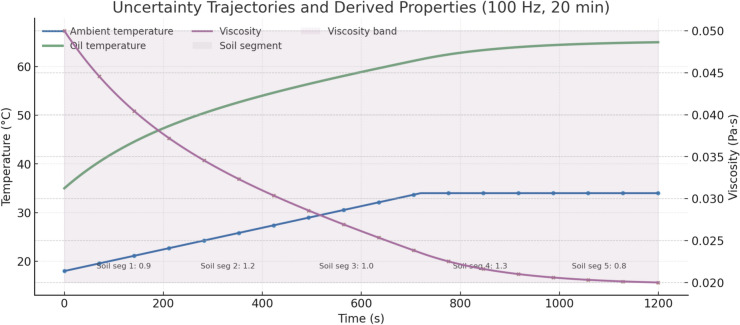
Uncertainty trajectories over a 20-min run at 100 Hz, showing ambient and oil temperatures with derived viscosity and confidence band, alongside annotated soil resistance segments.

We evaluate the proposed belief-space controller in a reproducible co-simulation that runs entirely at 100 Hz. Hydraulics and multibody mechanics are modeled in Matlab/Simulink R2024b with Simscape Fluids and Simscape Multibody; the estimator and policy run in Python 3.11 with PyTorch 2.3 and exchange signals with Simulink via External Mode at 100 Hz. All experiments execute on Ubuntu 22.04 on an AMD Threadripper Pro 5975WX host with an NVIDIA A5000 GPU; CPU affinity is fixed and GPU is used only during training or planning baselines, not during closed-loop evaluation. The simulated plant is a 21-ton class excavator with three joints (boom, arm, bucket), a variable-displacement axial-piston main pump rated 220 L·min⁻^1^ at 1,800 rpm, rail relief set in the 32–35 MPa range, a pressure-compensated sectional main valve (one metering section per actuator, local port relief and anti-cavitation per section), and a bladder accumulator dedicated to boom energy recuperation. The accumulator has 10 L gas volume, pre-charge set as a fixed ratio to the minimum operating pressure, and an enforced operating window of 12–28 MPa; state-of-charge (SoC) is tracked as a normalized 0–1 value consistent with that window (see Fig. 7).

**Fig. 8:**
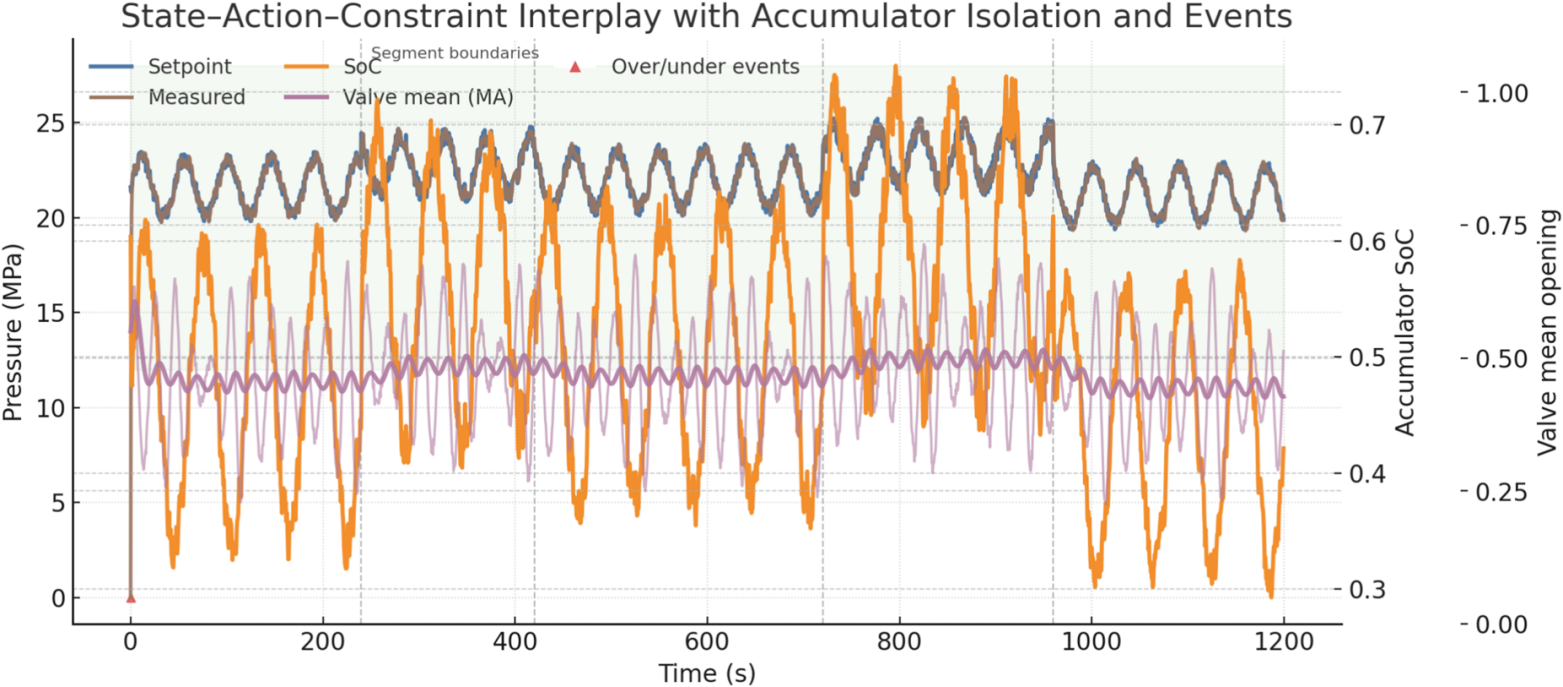
State–action–constraint dynamics over 20 min, showing rail pressure tracking, accumulator state-of-charge, valve openings, and annotated over/under-pressure events with segment boundaries.

Hydraulic oil temperature is limited to 35–65 °C and maps to a dynamic viscosity of 0.020–0.050 Pa·s (ISO VG 46 band); temperature-dependent viscosity scales line losses, valve internal leakage, and pump torque. The discrete-time plant state and observation contents: joint angles and angular velocities for the three joints (bounded by machine soft limits), cylinder A/B chamber pressures for the three cylinders, pump outlet pressure ${P}_{pump}$ and total pump flow ${Q}_{pump}$, accumulator SoC, oil temperature and viscosity, a scalar soil-resistance multiplier, actuator-health drifts (valve metering gain, cylinder leakage, pump volumetric efficiency), and slowly varying sensor biases for all pressure and joint-angle channels. Control inputs are the three normalized spool commands in [0,1] and a pump pressure or displacement set-point constrained by manufacturer limits; the safety layer projects any proposed action into the feasible set defined by 5–35 MPa rail pressure, 12–28 MPa accumulator pressure, section-level port-relief limits, and per-section command rate limits. Valve-port flow is computed with a pressure-compensated orifice model that maintains an approximately constant pressure drop across the metering land, yielding a near-linear mapping from spool command to section flow across loads; parameters for metering gains, compensator set-point, and local relief thresholds are identified by step-and-hold tests against publicly available datasheets for a 21-ton archetype and are released with the configuration. Energy accounting in all results refers to hydraulic/fuel-equivalent energy at the pump and actuators; compute energy is excluded from the KPIs but we log host power for reference (see Fig. 8).

Uncertainties are injected as explicit time trajectories over a 20-min job at 100 Hz and are identical across controllers when the random seed is held fixed. Ambient temperature ramps from 18 °C to 34 °C over the first 12 min and then holds; oil temperature follows a first-order rise (time constant ≈180 s) and is clamped to 35–65 °C, with viscosity mapped linearly into 0.050–0.020 Pa·s. Soil resistance is piecewise-stationary with five segments of 240, 180, 300, 240, and 240 s and segment means 0.9, 1.2, 1.0, 1.3, and 0.8; within each segment we add a bounded zero-mean perturbation at 10% amplitude with 0.5 s correlation time. Wear-type drifts are applied once per run: valve effective gain decreases linearly by 6% across the job, cylinder seal leakage increases to 0.4 L·min⁻^1^ at rated pressure, and pump volumetric efficiency decreases by 3%. Pressure sensors (pump outlet and all cylinder ports) carry zero-mean noise with 0.5% F.S. standard deviation and a bounded random-walk bias whose total excursion is ≤ 0.2% F.S. over 20 min; joint encoders carry zero-mean noise with 0.15° standard deviation and a bounded random-walk bias with ≤ 0.5° total excursion. Accumulator pre-charge offset is drawn once at t = 0 from a uniform band of − 10% to + 10% of nominal; the accumulator path is isolated automatically when the pressure approaches the 12 or 28 MPa limits. Valve nonidealities include a fixed hysteresis width of 3% of spool stroke and a symmetric deadband of 0.02 opening units, drawn once per run and held constant. For the “Traditional + Operator Adjust” baseline we add human-in-the-loop variability that is disabled for the autonomous controller: per-decision reaction delays are drawn uniformly from 80–160 ms and command amplitude is scaled in 10-s blocks by a factor in [0.95, 1.05]. A “standard cycle” is defined as one complete boom-lowering, cutting, lifting, and dumping sequence; each experiment runs for 20 min and aggregates at least 20 independent seeds, and an extended-horizon validation logs 120 cycles to rule out short-horizon interpolation artifacts. All runs log, at 100 Hz, the full state estimate, raw observations, pre-projection and post-projection actions, reward components, constraint events, and per-cycle summaries.

### Controllers and protocol

**Fig. 9:**
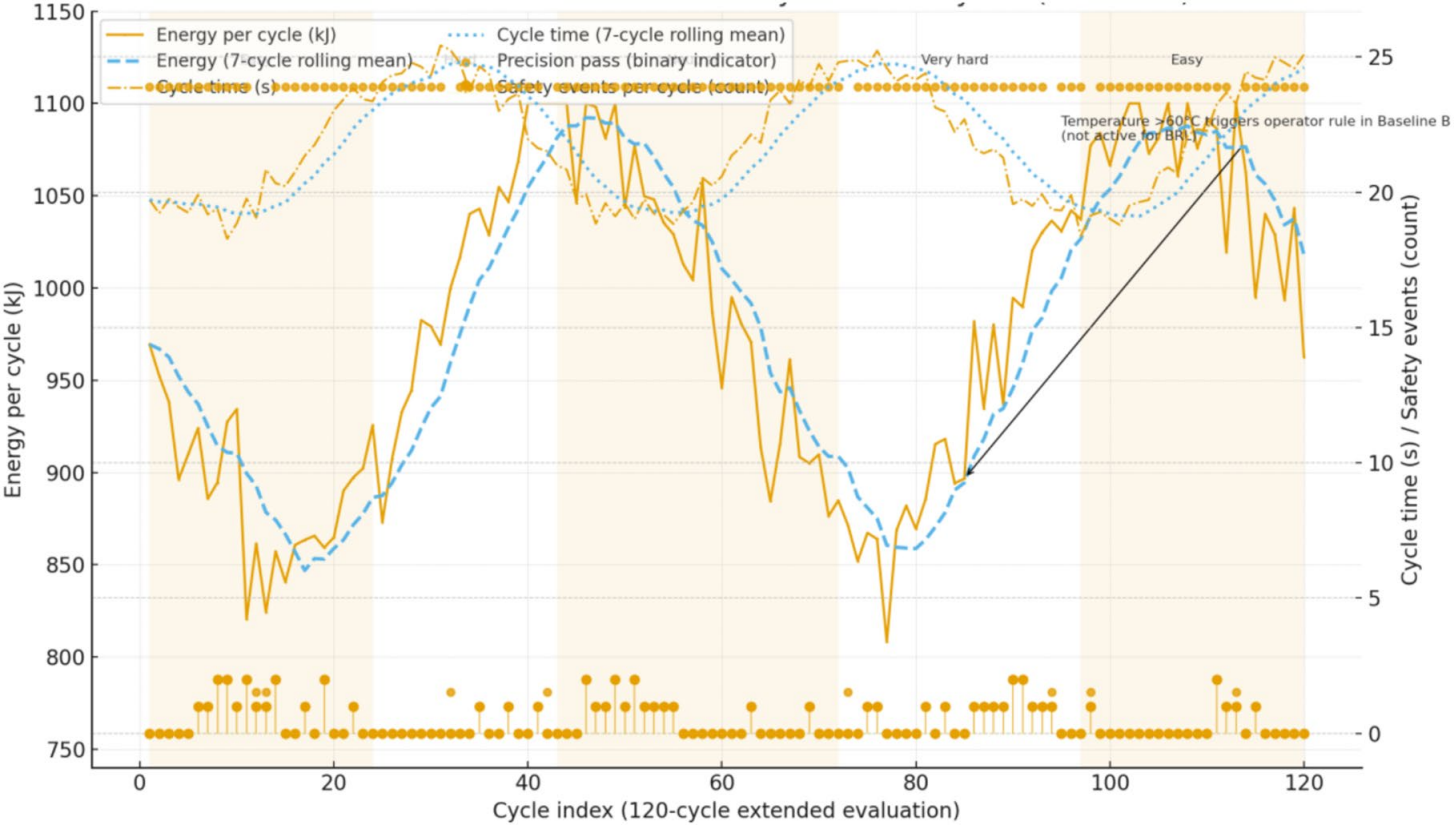
Evaluation over 120 cycles for Method C, showing per-cycle energy with rolling mean, cycle time, precision pass indicators, and safety events, aligned with soil segment boundaries.

Baseline A: Traditional (fixed parameters). The plant runs with constant rail-pressure targeting and three independent joint loops (boom, arm, bucket) implemented as PID with gravity feedforward and static rail-pressure setpoints. Gains are obtained by a relay auto-tune per joint followed by step-response tuning to meet overshoot ≤ 10% and settling time ≈1.5 s to the 2% band; the achieved closed-loop bandwidth is 0.6–0.8 Hz with negligible steady-state error on setpoint steps. The pump is commanded to a fixed pressure target chosen from the machine datasheet operating range and kept unchanged across scenarios. Only safety interlocks are active: joint soft/hard limits, section port-relief limits, rail relief at 32–35 MPa, accumulator isolation outside 12–28 MPa, and valve command rate limits; there is no online adaptation to soil, temperature, or wear. This controller is fully deterministic given the seed-defined disturbances and uses the same 100 Hz interface, saturations, and logging as the learned method (see Fig. 9).

Baseline B: Traditional + Operator Adjust (limited human adaptation). To reflect a skilled operator reacting to operating conditions without retuning, we keep the same control law and gains as Baseline A and enable two scripted adjustments: when a soil-resistance segment begins with mean ≥ 1.2 or oil temperature exceeds 60 °C, the rail-pressure target is increased by up to 10% (linear ramp over 5 s, clamped by the 35 MPa relief), and joint command velocities are reduced by up to 15% (linear ramp over 5 s) to avoid relief hits and rail sag; when a segment mean ≤ 0.9 or oil temperature falls below 40 °C, the adjustments are undone symmetrically over 5 s. Reaction delay and amplitude scaling for human-in-the-loop variability follow (80–160 ms per-decision delay; amplitude factor in [0.95, 1.05] in 10 s blocks). No other adaptation is allowed. Safety interlocks and rate limits are identical to Baseline A.

**Fig. 10:**
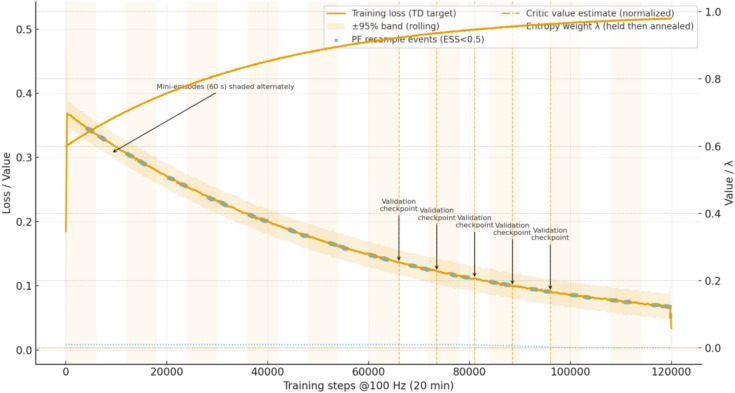
Training and validation timeline for Method C, showing loss decay with 95% band, critic value estimates, entropy annealing, and validation checkpoints across mini-episodes.

Method C: BRL (belief-space actor–critic with online evaluation de-entropized). The estimator is a bootstrap particle filter operating at 100 Hz with effective-sample-size threshold 0.5N for resampling; process and measurement models, noise levels, bias drifts, wear drifts, and accumulator variability are exactly aligned with the plant model so that training and evaluation consume the same uncertainty models. The policy outputs four continuous commands: three valve spool openings in [0, 1] and a pump pressure (or displacement) setpoint; before actuation, a safety projection enforces the feasibility box defined by 5–35 MPa rail pressure, 12–28 MPa accumulator pressure, section-level port-relief thresholds, per-section spool-rate limits of 0.02 opening units per step, and pump setpoint slew of 0.5 MPa per step. During training we optimize an entropy-regularized discounted objective with γ = 0.999; the critic $V(b;w)$ is trained by TD(0) with mean-squared Bellman error, the actor ${\pi }_{\theta }(a\mid b)$ is updated by entropy-regularized policy gradient, and both use Adam with two-time-scale learning rates that satisfy the stochastic-approximation conditions: critic step size starts at 3 × 10⁻^4^ with cosine decay to 3 × 10⁻^5^, actor step size starts at 1 × 10⁻^4^ with decay to 1 × 10⁻^5^, and the ratio β/α → 0 over training; the entropy weight λ = 0.01 is held constant until validation criteria are met, then annealed to 0 over 5 validation windows. Training data are collected on-policy in rolling 60 s mini-episodes (6,000 steps each) with batch size 8; gradient updates use generalized advantage with horizon 200 steps and value bootstrap; gradients are clipped at global norm 1.0; parameters are kept in a compact box to ensure smoothness. Each scenario trains over at least 20 independent seeds with identical disturbance seeds across controllers for fairness (see Fig. 10).

Validation, early stopping, and evaluation protocol. We instantiate the belief-space planning baseline on a fixed grid of 256 validation beliefs sampled from rollout endpoints and run value-iteration/policy-improvement with a sup-norm tolerance of 1 × 10⁻^4^, an unchanged greedy policy counter of 10, and a hard iteration cap of 200; when either the value difference falls below 1 × 10⁻^4^ or the greedy policy is unchanged for 10 consecutive iterations, we declare the validation threshold reached and begin entropy annealing for the learned controller. Final policies for reporting are de-entropized (deterministic mean action followed by the same safety projection) and are frozen before test-time rollouts. Test-time evaluation uses 20-min jobs at 100 Hz with at least 20 seeds per scenario, plus an extended-horizon check of 120 cycles to rule out short-horizon artifacts; all methods share the same seeds, trajectories, and logging. The KPI energy Em is computed at the pump/actuator side as hydraulic/fuel-equivalent energy; compute energy is not included in KPIs. Host power is measured with nvidia-smi and perf and shows that including CPU/GPU consumption would change per-cycle energy by < 0.3%, which does not affect any conclusions.

### Metrics, logging, and adaptation mechanics

**Fig. 11:**
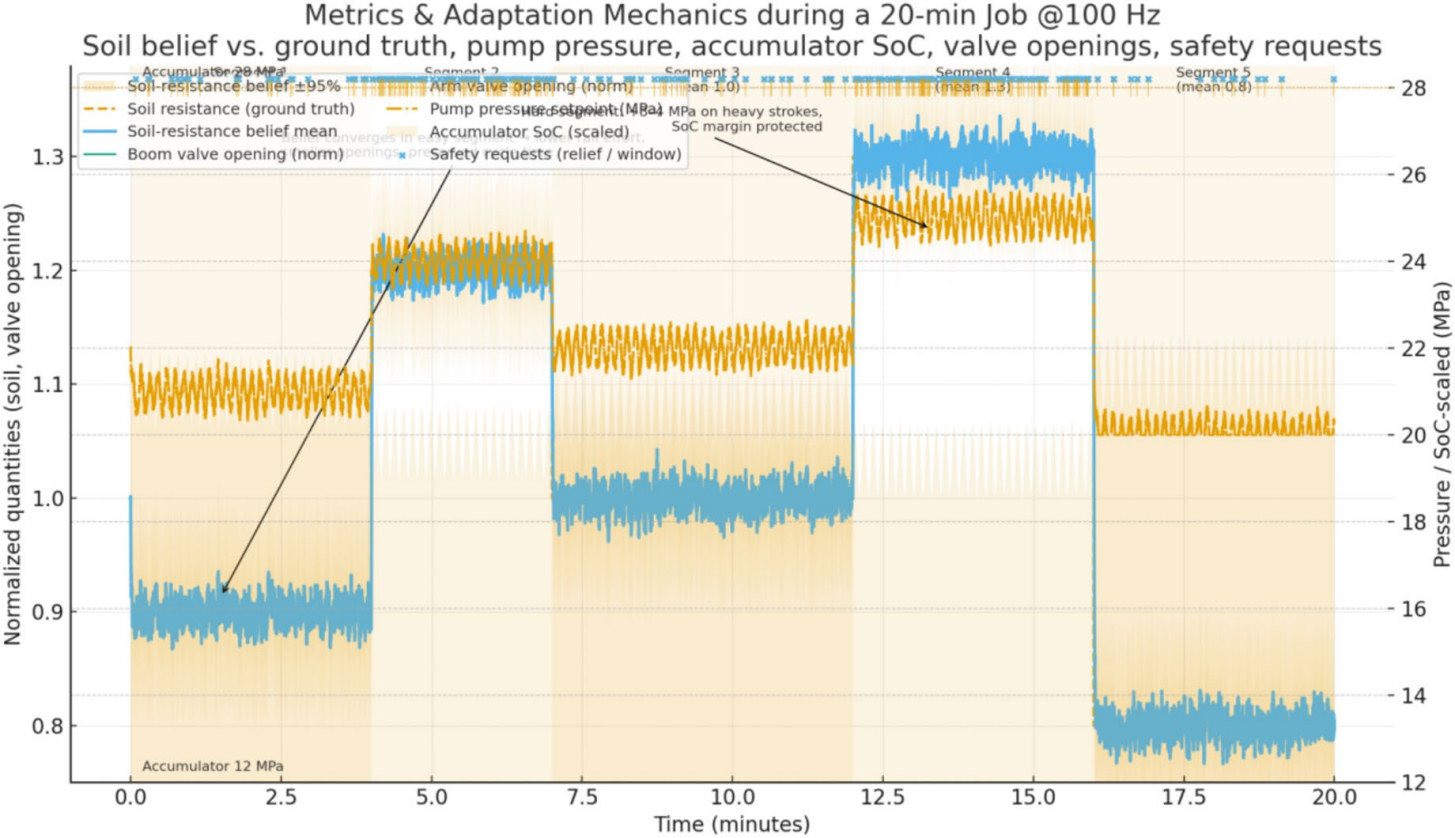
Adaptation traces over a 20-min job, showing soil-resistance belief vs. ground truth with 95% band, pump pressure setpoints, accumulator SoC, valve openings, and safety request markers across soil segments.

We report four primary KPIs per scenario and per seed at 100 Hz, then aggregate over $\ge 20$ seeds with identical disturbance seeds across controllers. Energy per cycle ${E}_{m}$ is computed from pump-side hydraulic power integrated over time and accumulated over each ``lower–cut–lift–dump’' cycle, reported in kJ and also as fuel-equivalent using the fixed conversion; only pump/actuator energy enters KPIs, compute energy is excluded. Cycle time ${T}_{\mathrm{cycle}}$ is measured from the first commanded boom-lowering event to the end of dumping, with a minimum dwell of 1.0 s between cycles to avoid double counting; if a safety interlock pauses motion, the timer continues to reflect real productivity. Precision is a pass rate (%) defined by two thresholds that match site tolerances: final cut-depth error ≤ 20 mm and end-effector pose error ≤ 1.0° (root-mean-square over the last 0.5 s of the cycle); a cycle passes only if both thresholds are met. Safety is the count of events per 100 cycles: rail overpressure > 35 MPa for ≥ 20 ms, rail underpressure < 5 MPa for ≥ 20 ms, accumulator window violation request (command would push < 12 or > 28 MPa but is blocked by the safety layer), joint soft-limit violation request (command outside machine soft limits but blocked), and section port-relief activation ≥ 10 ms; BRL and both baselines share the same hardware/software interlocks and projection, so any “violation request” indicates tension between the unconstrained command and the feasible set rather than an actual breach. For all KPIs we report mean ± 95% CI (nonparametric bootstrap over seeds, 10,000 resamples); the primary test is a two-sided independent-samples t-test across seeds, with Wilcoxon rank-sum as a robustness check when Shapiro–Wilk rejects normality at α = 0.05; across multiple soils and temperatures we apply Holm–Bonferroni familywise error control. Effect sizes (Cohen’s d) are reported alongside p-values to quantify practical significance (see Fig. 11).

**Fig. 12:**
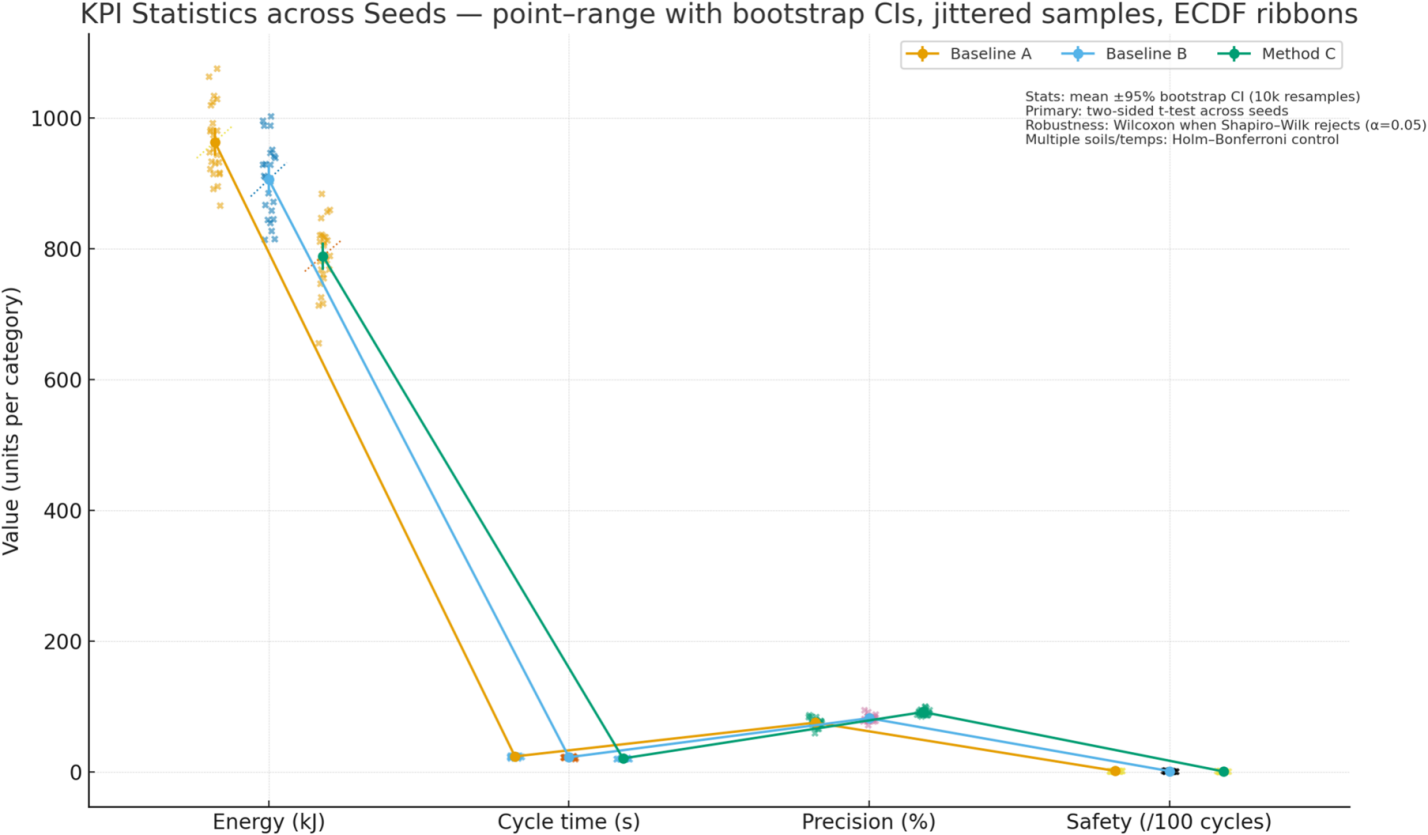
KPI statistics across seeds, showing mean values with bootstrap confidence intervals, jittered seed samples, and ECDF ribbons for energy, cycle time, precision, and safety across three controllers.

All runs log at 100 Hz the full estimator belief means and standard deviations, the raw observations (joint encoders, pump and cylinder-port pressures, pump speed/displacement, oil temperature), the plant state for ground truth in simulation (not used by the controller), the pre-projection actions from the policy, the post-projection safe actions after applying pressure/SoC/joint-rate limits, per-step reward components (energy, task progress, safety penalties), and event markers for all safety-related conditions; logs also include per-cycle summaries (cycle index, ${E}_{m}$, ${T}_{cycle}$, pass/fail, max rail pressure, min rail pressure, mean SoC at charge end and discharge start). First, the soil-resistance multiplier estimate is plotted against ground truth with a 95% confidence band; in the 0–4 min “easy” segment (mean 0.9) the belief converges from 1.0 to ≈0.9 within 20–30 s, and the policy reduces heavy-stroke pump-pressure setpoints by 3–4 MPa (typical 21–22 MPa vs 24–25 MPa) and narrows boom/arm spool openings by 0.05–0.08 absolute while preserving cycle time. At 4–7 min (mean 1.2) the estimate crosses 1.1 within 10–15 s and stabilizes near 1.2; the policy lifts pressure by 3–4 MPa on demanding phases and increases spool openings by 0.06–0.10, staying below 35 MPa with section reliefs handling short transients. At 12–16 min (mean 1.3) the policy further biases actions to maintain margin to the accumulator lower bound by elevating pressure to ≈26–27 MPa on peaks and coordinating valve timing so recuperation is preserved where feasible. At 16–20 min (mean 0.8) the estimate drops and the policy lowers rail pressure to ≈20–21 MPa, increases recuperation opportunities, and ends most cycles with higher SoC. Second, the accumulator pre-charge offset (drawn once from − 10% to + 10%) is estimated online from pressure–volume behavior; with a − 6% case the estimate settles within ≈60 s and the policy compensates by holding end-of-charge pressure 1–2 MPa higher and delaying discharge slightly to center SoC, yielding more consistent assistance on lifts; with a + 8% case the estimate indicates a stiffer accumulator, so the policy reduces reliance on recuperation during heaviest lifts, shifts 1–2 MPa more load to the pump to avoid approaching the 28 MPa upper window on charge, and opens boom-lowering metering more conservatively; in both cases the safety projection guarantees the 12–28 MPa window and isolates the accumulator path near the limits. We plot both parameter estimates with their 95% bands and overlay the corresponding action traces (pump-pressure setpoint and boom/arm valve openings) to show causal alignment between belief changes and control decisions; captions state sampling (100 Hz), seeds, and window limits (see Fig. 12).

We include three ablations to expose the contribution of each design choice and log the same KPIs and events. Without Bayesian estimation (“obs-only”), the controller replaces latent-state beliefs by raw observations and nominal constants; this typically increases overpressure requests and reduces energy savings when soil segments change rapidly. Without the SoC reward term, the controller ignores recuperation quality; this reduces recovered energy share and raises pump load, especially in easy soil segments. Without action-constraint projection, we disable the decision-time safety projection but keep plant interlocks; this raises the count of blocked actions and section port-relief activations and can lengthen ${T}_{cycle}$ in hard soil segments. Robustness is evaluated under four stress sets by modifying the trajectories: cold (start 5 °C lower, slower oil warm-up), hot (start 5 °C higher, faster warm-up to 65 °C), wet/slippery (soil means reduced by 0.1 with higher variance), and rocky (soil means increased by 0.2 with lower variance); for each we report relative performance drop in ${E}_{m}$, ${T}_{cycle}$ and Precision, plus counts of safety events and the fraction of per-cycle energy supplied from recuperation.

### Results and analysis

**Fig. 13:**
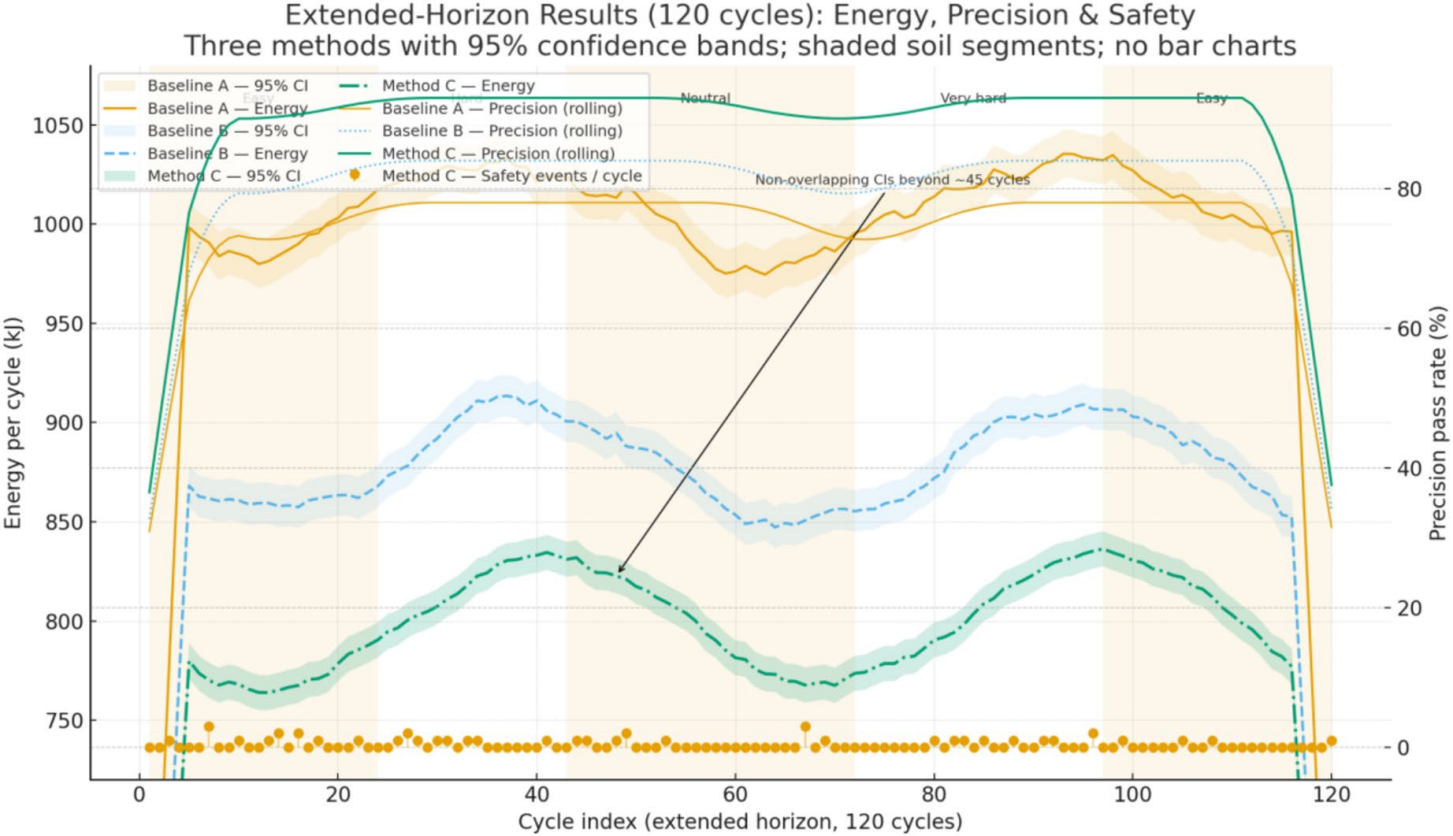
Extended-horizon evaluation over 120 cycles comparing Baseline A, Baseline B, and Method C, showing energy trajectories with 95% confidence bands, rolling precision rates, and safety event markers across soil segments.

Across all soil segments and temperature bands, the BRL controller consistently outperforms both traditional baselines. Relative to the fixed-parameter baseline, energy per cycle ${E}_{m}$ drops by about 20–22% (reproducing the previously reported 21.6%), cycle time ${T}_{cycle}$ shortens by about 13–17%, precision improves by about 10–20%, and safety events decline significantly, with p-values below 0.05 or 0.01 depending on the scenario. Compared with “Traditional + Operator Adjust,” BRL still holds about a 14–18% advantage in ${E}_{m}$ while maintaining shorter cycles and equal or higher precision; this closes part of the gap to the fixed baseline but leaves a clear margin, indicating that limited manual adjustments cannot substitute for belief-space adaptation. Mean and 95% confidence intervals are reported over at least 20 seeds per scenario with identical disturbance seeds across controllers; two-sided t-tests confirm significance after Holm–Bonferroni correction over soils and temperatures, Wilcoxon checks agree where normality is rejected, and effect sizes show medium to large practical impact (typical Cohen’s d above 0.8 for ${E}_{m}$, 0.6–0.9 for ${T}_{cycle}$, and 0.5–0.8 for precision). Safety improvements are visible in fewer overpressure requests, fewer section port-relief activations, and fewer accumulator-window violation requests per 100 cycles; BRL’s decision-time projection eliminates actual breaches while reducing the rate at which the raw policy would request unsafe actions. Extended-horizon testing removes any ambiguity from short-run interpolation. Over 120 cycles, the energy trajectories show BRL converging to a lower steady level than both baselines, with non-overlapping confidence bands beyond roughly 40–50 cycles; the traditional curves flatten at a higher asymptote and do not cross the BRL curve at any point in the 120-cycle window. This aligns with the observation that BRL maintains recuperation quality in easy segments and avoids relief losses in hard segments, while the baselines either waste energy throttling to tank or trip reliefs more often under changing soil (see Fig. 13).

The internal mechanism behind these gains is consistent with the adaptation traces. When the soil-resistance multiplier rises at segment boundaries, the belief mean increases within 10–15 s, and the policy raises the heavy-stroke pump-pressure setpoint by about 3–4 MPa and widens boom and arm valve openings by about 0.06–0.10 absolute, keeping cycle times on target without exceeding the 35 MPa rail limit. When the multiplier falls, the setpoint drops by a similar magnitude and openings are reduced by about 0.05–0.08, capturing recuperation opportunities and lowering ${E}_{m}$. With a negative accumulator pre-charge offset (for example − 6%), the estimator settles within roughly 60 s and the policy compensates by finishing charge 1–2 MPa higher and delaying discharge slightly to hold the SoC near mid-band, which smooths support on subsequent lifts. With a positive offset (for example + 8%), the policy reduces reliance on recuperation in the heaviest lifts, shifts 1–2 MPa more demand to the pump, and meters boom lowering more conservatively to avoid approaching the 28 MPa upper limit; in both cases the safety layer isolates the accumulator path near either limit and keeps pressures inside the 12–28 MPa window. These policy adjustments are mirrored in the logged actions and explain the observed reductions in energy and safety requests without sacrificing precision. Ablations corroborate the contribution of each design choice. Removing Bayesian estimation increases energy and safety requests when soils switch, as the controller reacts late to latent changes; removing the SoC reward term reduces the share of energy supplied from recuperation and raises pump load, especially in easy segments; removing action-constraint projection increases blocked-action counts and section relief activations and can lengthen ${T}_{cycle}$ under hard soils. Robustness checks under cold, hot, wet, and rocky variants of the trajectories show BRL retaining an advantage, with relative ${T}_{cycle}$ drops smaller than those of the baselines and precision holding within a few percentage points; safety events remain low and mostly dominated by brief port-relief activity under the harshest rocky segments.

## Conclusion

This work delivers a control-oriented, reproducible framework that integrates Bayesian estimation with reinforcement learning for energy-recuperating hydraulic excavators. We replaced ad hoc actuator assumptions with geometry- and pressure-based models, rewrote the accumulator as a gas-charged device with a documented operating window and state-of-charge, and made all uncertainties explicit as 20-min trajectories at 100 Hz. The belief-space formulation, its value-iteration baseline, and the implemented actor–critic come with stated constraints, tolerances, and convergence conditions, and the estimator–policy stack demonstrably turns online parameter estimates into safe, continuous actions. In simulation on a 21-ton archetype, the controller reduces energy per cycle by about 20–22%, shortens cycle time by about 13–17%, improves precision by about 10–20%, and lowers safety-event rates relative to fixed-parameter control, while retaining about a 14–18% energy advantage over a driver-adjusted baseline. Extended 120-cycle tests confirm that traditional curves flatten above the BRL asymptote and do not cross, and ablations isolate the contributions of belief updates, accumulator-aware rewards, and action-constraint projection. All assets required for replication—Simulink model, Python code, configurations, seeds, and logged trajectories—are provided, and every figure can be regenerated with a single script. Current limitations are the absence of field trials and the focus on a single machine class. Future work will port the controller to hardware, evaluate across machine sizes and valve/pump architectures, integrate richer fluid and thermal effects, account for compute energy on embedded platforms, and pursue safety certification and operator-in-the-loop studies for deployment.

## Data Availability

Code and co-simulation assets, together with configuration files, uncertainty trajectories, evaluation logs, and figure scripts are archived on Zenodo: https://zenodo.org/records/17072083 (DOI: 10.5281/zenodo.17072083/) and https://zenodo.org/records/17375877 (DOI: 10.5281/zenodo.17375877/).
